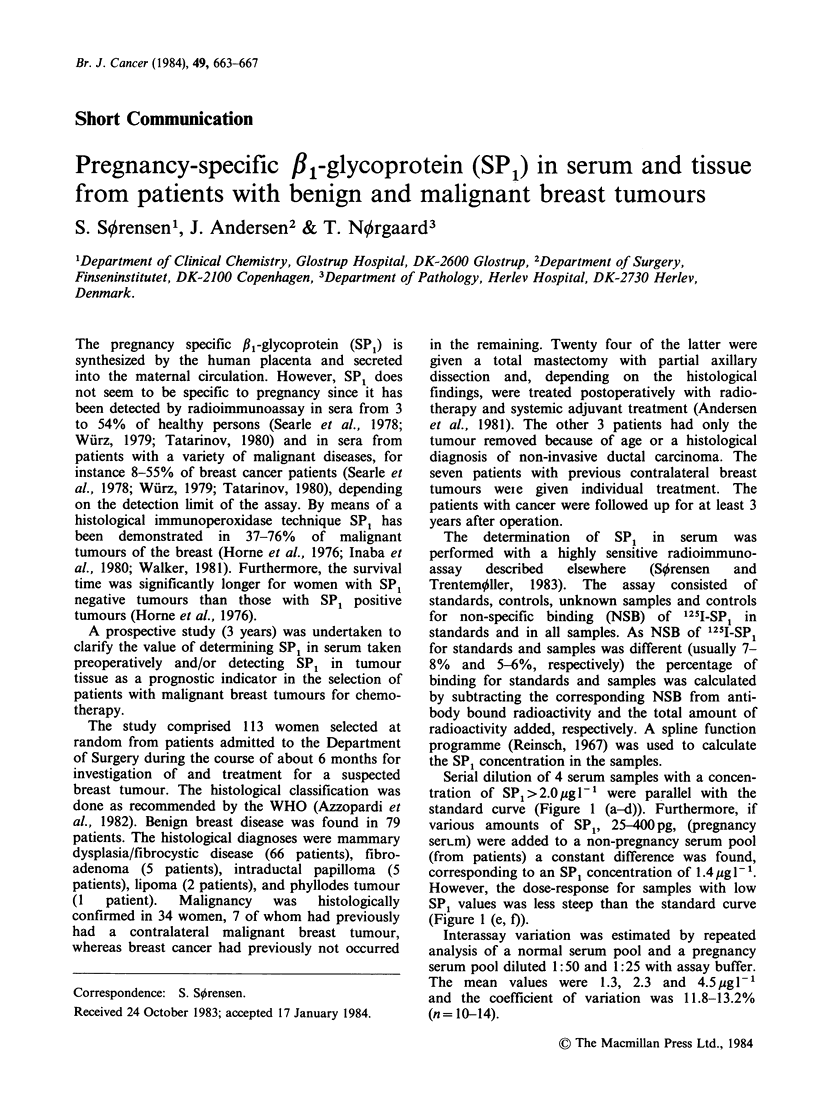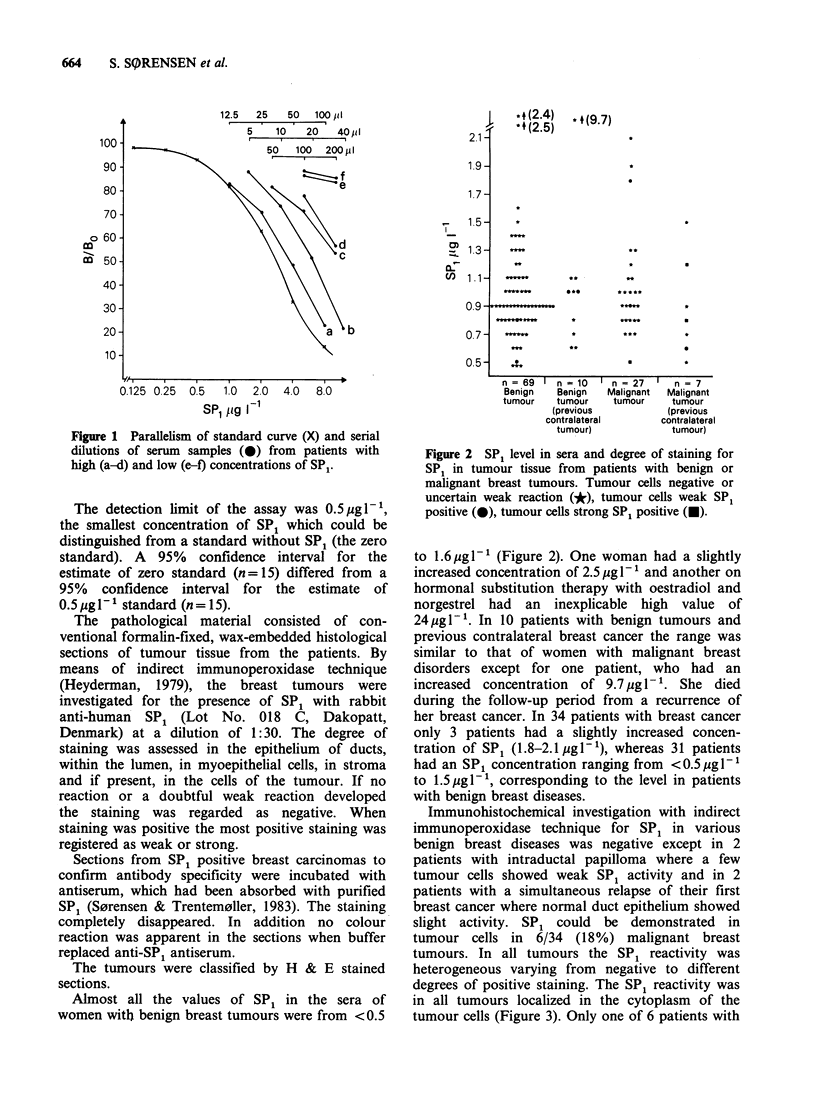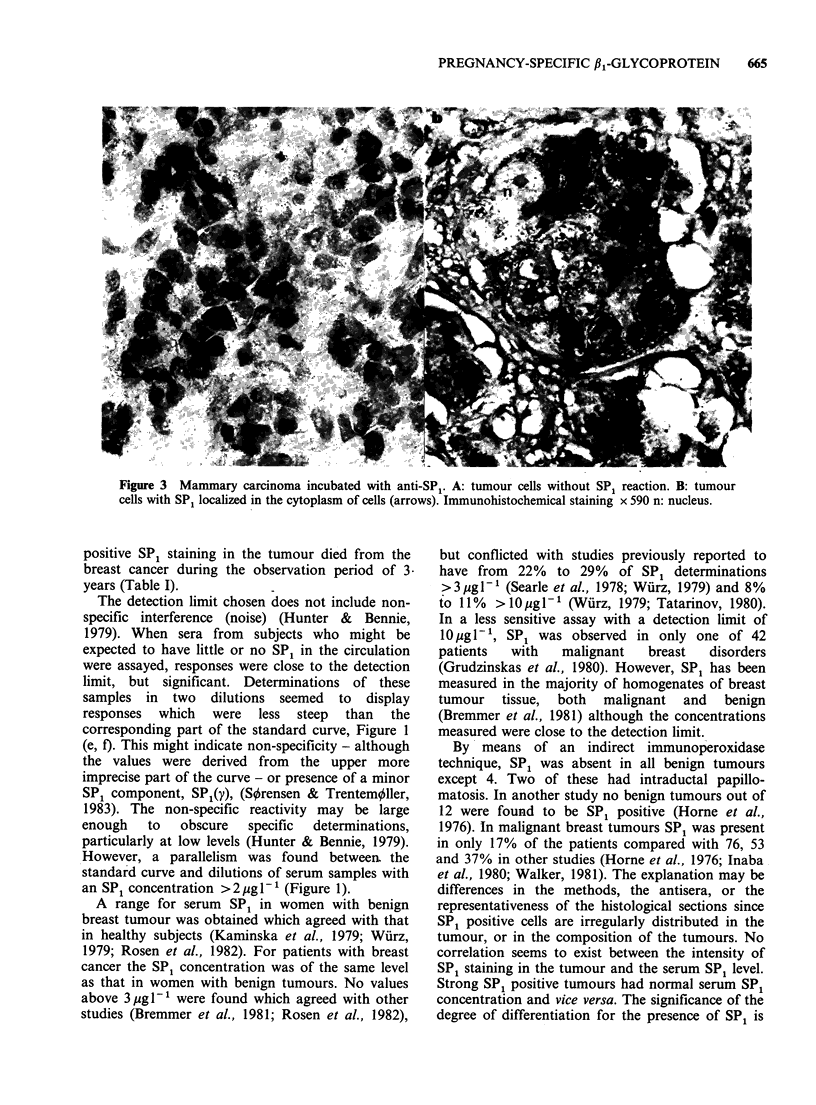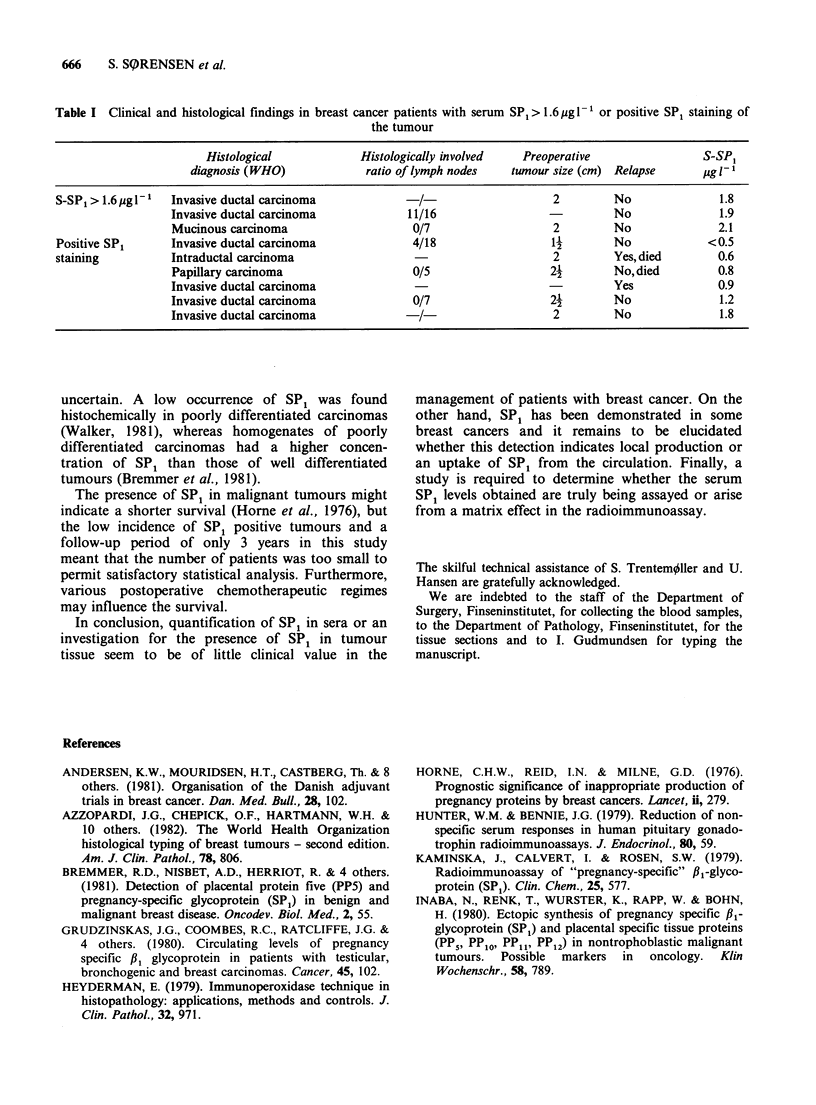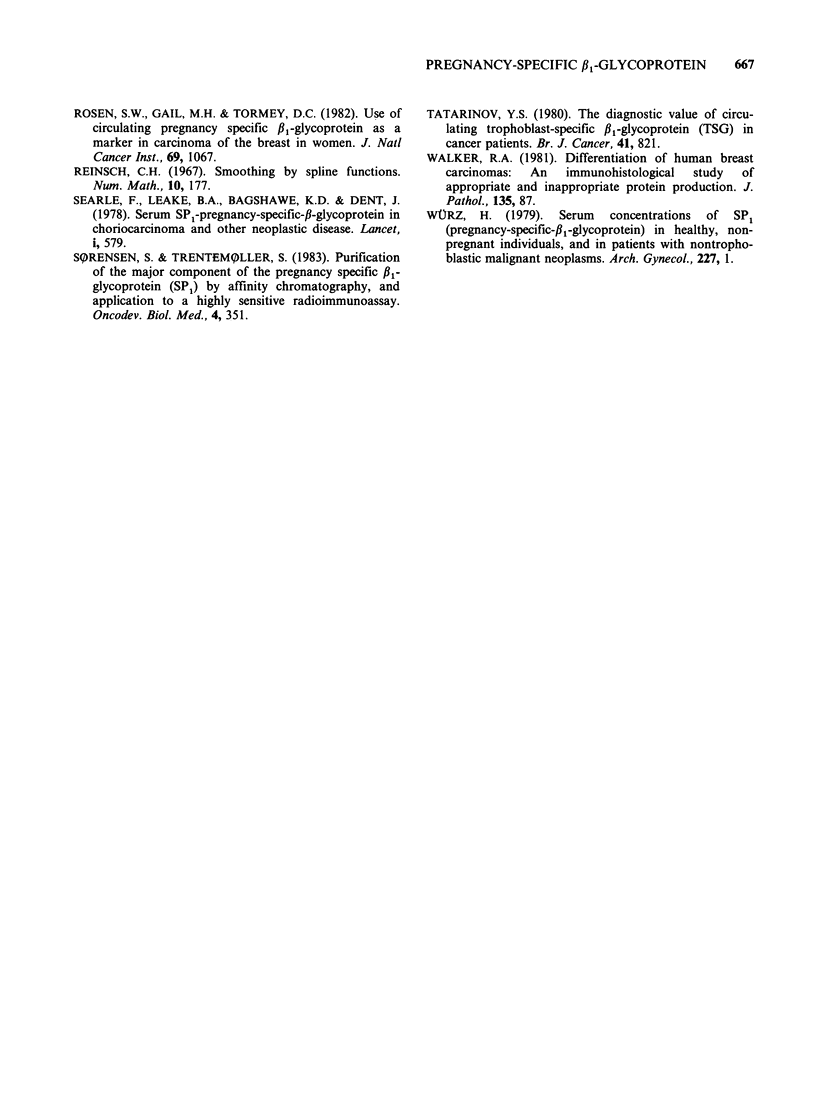# Pregnancy-specific beta 1-glycoprotein (SP1) in serum and tissue from patients with benign and malignant breast tumours.

**DOI:** 10.1038/bjc.1984.103

**Published:** 1984-05

**Authors:** S. Sørensen, J. Andersen, T. Nørgaard

## Abstract

**Images:**


Br. J. Cancer (1984), 49, 663–667

### Short Communication

### Pregnancy-specific $\beta_1$-glycoprotein ($SP_1$) in serum and tissue from patients with benign and malignant breast tumours

S. Sørensen[1], J. Andersen[2] & T. Nørgaard[3]

[1]*Department of Clinical Chemistry, Glostrup Hospital, DK-2600 Glostrup,* [2]*Department of Surgery, Finseninstitutet, DK-2100 Copenhagen,* [3]*Department of Pathology, Herlev Hospital, DK-2730 Herlev, Denmark.*

The pregnancy specific $\beta_1$-glycoprotein ($SP_1$) is synthesized by the human placenta and secreted into the maternal circulation. However, $SP_1$ does not seem to be specific to pregnancy since it has been detected by radioimmunoassay in sera from 3 to 54% of healthy persons (Searle *et al.*, 1978; Würz, 1979; Tatarinov, 1980) and in sera from patients with a variety of malignant diseases, for instance 8–55% of breast cancer patients (Searle *et al.*, 1978; Würz, 1979; Tatarinov, 1980), depending on the detection limit of the assay. By means of a histological immunoperoxidase technique $SP_1$ has been demonstrated in 37–76% of malignant tumours of the breast (Horne *et al.*, 1976; Inaba *et al.*, 1980; Walker, 1981). Furthermore, the survival time was significantly longer for women with $SP_1$ negative tumours than those with $SP_1$ positive tumours (Horne *et al.*, 1976).

A prospective study (3 years) was undertaken to clarify the value of determining $SP_1$ in serum taken preoperatively and/or detecting $SP_1$ in tumour tissue as a prognostic indicator in the selection of patients with malignant breast tumours for chemotherapy.

The study comprised 113 women selected at random from patients admitted to the Department of Surgery during the course of about 6 months for investigation of and treatment for a suspected breast tumour. The histological classification was done as recommended by the WHO (Azzopardi *et al.*, 1982). Benign breast disease was found in 79 patients. The histological diagnoses were mammary dysplasia/fibrocystic disease (66 patients), fibroadenoma (5 patients), intraductal papilloma (5 patients), lipoma (2 patients), and phyllodes tumour (1 patient). Malignancy was histologically confirmed in 34 women, 7 of whom had previously had a contralateral malignant breast tumour, whereas breast cancer had previously not occurred in the remaining. Twenty four of the latter were given a total mastectomy with partial axillary dissection and, depending on the histological findings, were treated postoperatively with radiotherapy and systemic adjuvant treatment (Andersen *et al.*, 1981). The other 3 patients had only the tumour removed because of age or a histological diagnosis of non-invasive ductal carcinoma. The seven patients with previous contralateral breast tumours weie given individual treatment. The patients with cancer were followed up for at least 3 years after operation.

The determination of $SP_1$ in serum was performed with a highly sensitive radioimmunoassay described elsewhere (Sørensen and Trentemøller, 1983). The assay consisted of standards, controls, unknown samples and controls for non-specific binding (NSB) of $^{125}I$-$SP_1$ in standards and in all samples. As NSB of $^{125}I$-$SP_1$ for standards and samples was different (usually 7–8% and 5–6%, respectively) the percentage of binding for standards and samples was calculated by subtracting the corresponding NSB from antibody bound radioactivity and the total amount of radioactivity added, respectively. A spline function programme (Reinsch, 1967) was used to calculate the $SP_1$ concentration in the samples.

Serial dilution of 4 serum samples with a concentration of $SP_1 > 2.0\,\mu g\,l^{-1}$ were parallel with the standard curve (Figure 1 (a–d)). Furthermore, if various amounts of $SP_1$, 25–400 pg, (pregnancy serum) were added to a non-pregnancy serum pool (from patients) a constant difference was found, corresponding to an $SP_1$ concentration of $1.4\,\mu g\,l^{-1}$. However, the dose-response for samples with low $SP_1$ values was less steep than the standard curve (Figure 1 (e, f)).

Interassay variation was estimated by repeated analysis of a normal serum pool and a pregnancy serum pool diluted 1:50 and 1:25 with assay buffer. The mean values were 1.3, 2.3 and $4.5\,\mu g\,l^{-1}$ and the coefficient of variation was 11.8–13.2% ($n = 10$–$14$).

Correspondence: S. Sørensen.

Received 24 October 1983; accepted 17 January 1984.

© The Macmillan Press Ltd., 1984

**Figure 1** Parallelism of standard curve (X) and serial dilutions of serum samples (●) from patients with high (a–d) and low (e–f) concentrations of $SP_1$.

**Figure 2** $SP_1$ level in sera and degree of staining for $SP_1$ in tumour tissue from patients with benign or malignant breast tumours. Tumour cells negative or uncertain weak reaction (★), tumour cells weak $SP_1$ positive (●), tumour cells strong $SP_1$ positive (■).

The detection limit of the assay was $0.5\,\mu g\,l^{-1}$, the smallest concentration of $SP_1$ which could be distinguished from a standard without $SP_1$ (the zero standard). A 95% confidence interval for the estimate of zero standard ($n=15$) differed from a 95% confidence interval for the estimate of $0.5\,\mu g\,l^{-1}$ standard ($n=15$).

The pathological material consisted of conventional formalin-fixed, wax-embedded histological sections of tumour tissue from the patients. By means of indirect immunoperoxidase technique (Heyderman, 1979), the breast tumours were investigated for the presence of $SP_1$ with rabbit anti-human $SP_1$ (Lot No. 018 C, Dakopatt, Denmark) at a dilution of 1:30. The degree of staining was assessed in the epithelium of ducts, within the lumen, in myoepithelial cells, in stroma and if present, in the cells of the tumour. If no reaction or a doubtful weak reaction developed the staining was regarded as negative. When staining was positive the most positive staining was registered as weak or strong.

Sections from $SP_1$ positive breast carcinomas to confirm antibody specificity were incubated with antiserum, which had been absorbed with purified $SP_1$ (Sørensen & Trentemøller, 1983). The staining completely disappeared. In addition no colour reaction was apparent in the sections when buffer replaced anti-$SP_1$ antiserum.

The tumours were classified by H & E stained sections.

Almost all the values of $SP_1$ in the sera of women with benign breast tumours were from <0.5 to $1.6\,\mu g\,l^{-1}$ (Figure 2). One woman had a slightly increased concentration of $2.5\,\mu g\,l^{-1}$ and another on hormonal substitution therapy with oestradiol and norgestrel had an inexplicable high value of $24\,\mu g\,l^{-1}$. In 10 patients with benign tumours and previous contralateral breast cancer the range was similar to that of women with malignant breast disorders except for one patient, who had an increased concentration of $9.7\,\mu g\,l^{-1}$. She died during the follow-up period from a recurrence of her breast cancer. In 34 patients with breast cancer only 3 patients had a slightly increased concentration of $SP_1$ ($1.8$–$2.1\,\mu g\,l^{-1}$), whereas 31 patients had an $SP_1$ concentration ranging from $<0.5\,\mu g\,l^{-1}$ to $1.5\,\mu g\,l^{-1}$, corresponding to the level in patients with benign breast diseases.

Immunohistochemical investigation with indirect immunoperoxidase technique for $SP_1$ in various benign breast diseases was negative except in 2 patients with intraductal papilloma where a few tumour cells showed weak $SP_1$ activity and in 2 patients with a simultaneous relapse of their first breast cancer where normal duct epithelium showed slight activity. $SP_1$ could be demonstrated in tumour cells in 6/34 (18%) malignant breast tumours. In all tumours the $SP_1$ reactivity was heterogeneous varying from negative to different degrees of positive staining. The $SP_1$ reactivity was in all tumours localized in the cytoplasm of the tumour cells (Figure 3). Only one of 6 patients with

**Figure 3** Mammary carcinoma incubated with anti-$SP_1$. A: tumour cells without $SP_1$ reaction. **B**: tumour cells with $SP_1$ localized in the cytoplasm of cells (arrows). Immunohistochemical staining $\times 590$ n: nucleus.

positive $SP_1$ staining in the tumour died from the breast cancer during the observation period of 3 years (Table I).

The detection limit chosen does not include non-specific interference (noise) (Hunter & Bennie, 1979). When sera from subjects who might be expected to have little or no $SP_1$ in the circulation were assayed, responses were close to the detection limit, but significant. Determinations of these samples in two dilutions seemed to display responses which were less steep than the corresponding part of the standard curve, Figure 1 (e, f). This might indicate non-specificity – although the values were derived from the upper more imprecise part of the curve – or presence of a minor $SP_1$ component, $SP_1(\gamma)$, (Sørensen & Trentemøller, 1983). The non-specific reactivity may be large enough to obscure specific determinations, particularly at low levels (Hunter & Bennie, 1979). However, a parallelism was found between the standard curve and dilutions of serum samples with an $SP_1$ concentration $>2\,\mu g\,l^{-1}$ (Figure 1).

A range for serum $SP_1$ in women with benign breast tumour was obtained which agreed with that in healthy subjects (Kaminska *et al.*, 1979; Würz, 1979; Rosen *et al.*, 1982). For patients with breast cancer the $SP_1$ concentration was of the same level as that in women with benign tumours. No values above $3\,\mu g\,l^{-1}$ were found which agreed with other studies (Bremmer *et al.*, 1981; Rosen *et al.*, 1982), but conflicted with studies previously reported to have from 22% to 29% of $SP_1$ determinations $>3\,\mu g\,l^{-1}$ (Searle *et al.*, 1978; Würz, 1979) and 8% to 11% $>10\,\mu g\,l^{-1}$ (Würz, 1979; Tatarinov, 1980). In a less sensitive assay with a detection limit of $10\,\mu g\,l^{-1}$, $SP_1$ was observed in only one of 42 patients with malignant breast disorders (Grudzinskas *et al.*, 1980). However, $SP_1$ has been measured in the majority of homogenates of breast tumour tissue, both malignant and benign (Bremmer *et al.*, 1981) although the concentrations measured were close to the detection limit.

By means of an indirect immunoperoxidase technique, $SP_1$ was absent in all benign tumours except 4. Two of these had intraductal papillomatosis. In another study no benign tumours out of 12 were found to be $SP_1$ positive (Horne *et al.*, 1976). In malignant breast tumours $SP_1$ was present in only 17% of the patients compared with 76, 53 and 37% in other studies (Horne *et al.*, 1976; Inaba *et al.*, 1980; Walker, 1981). The explanation may be differences in the methods, the antisera, or the representativeness of the histological sections since $SP_1$ positive cells are irregularly distributed in the tumour, or in the composition of the tumours. No correlation seems to exist between the intensity of $SP_1$ staining in the tumour and the serum $SP_1$ level. Strong $SP_1$ positive tumours had normal serum $SP_1$ concentration and *vice versa*. The significance of the degree of differentiation for the presence of $SP_1$ is

**Table I** Clinical and histological findings in breast cancer patients with serum $SP_1 > 1.6 \mu g l^{-1}$ or positive $SP_1$ staining of the tumour

| | *Histological diagnosis (WHO)* | *Histologically involved ratio of lymph nodes* | *Preoperative tumour size (cm)* | *Relapse* | *S-SP₁* $\mu g l^{-1}$ |
|---|---|---|---|---|---|
| $S\text{-}SP_1 > 1.6 \mu g l^{-1}$ | Invasive ductal carcinoma | —/— | 2 | No | 1.8 |
| | Invasive ductal carcinoma | 11/16 | — | No | 1.9 |
| | Mucinous carcinoma | 0/7 | 2 | No | 2.1 |
| Positive $SP_1$ staining | Invasive ductal carcinoma | 4/18 | 1½ | No | <0.5 |
| | Intraductal carcinoma | — | 2 | Yes, died | 0.6 |
| | Papillary carcinoma | 0/5 | 2½ | No, died | 0.8 |
| | Invasive ductal carcinoma | — | — | Yes | 0.9 |
| | Invasive ductal carcinoma | 0/7 | 2½ | No | 1.2 |
| | Invasive ductal carcinoma | —/— | 2 | No | 1.8 |

uncertain. A low occurrence of $SP_1$ was found histochemically in poorly differentiated carcinomas (Walker, 1981), whereas homogenates of poorly differentiated carcinomas had a higher concentration of $SP_1$ than those of well differentiated tumours (Bremmer *et al.*, 1981).

The presence of $SP_1$ in malignant tumours might indicate a shorter survival (Horne *et al.*, 1976), but the low incidence of $SP_1$ positive tumours and a follow-up period of only 3 years in this study meant that the number of patients was too small to permit satisfactory statistical analysis. Furthermore, various postoperative chemotherapeutic regimes may influence the survival.

In conclusion, quantification of $SP_1$ in sera or an investigation for the presence of $SP_1$ in tumour tissue seem to be of little clinical value in the management of patients with breast cancer. On the other hand, $SP_1$ has been demonstrated in some breast cancers and it remains to be elucidated whether this detection indicates local production or an uptake of $SP_1$ from the circulation. Finally, a study is required to determine whether the serum $SP_1$ levels obtained are truly being assayed or arise from a matrix effect in the radioimmunoassay.

The skilful technical assistance of S. Trentemøller and U. Hansen are gratefully acknowledged.

We are indebted to the staff of the Department of Surgery, Finseninstitutet, for collecting the blood samples, to the Department of Pathology, Finseninstitutet, for the tissue sections and to I. Gudmundsen for typing the manuscript.

### References

ANDERSEN, K.W., MOURIDSEN, H.T., CASTBERG, Th. & 8 others. (1981). Organisation of the Danish adjuvant trials in breast cancer. *Dan. Med. Bull.*, **28**, 102.

AZZOPARDI, J.G., CHEPICK, O.F., HARTMANN, W.H. & 10 others. (1982). The World Health Organization histological typing of breast tumours – second edition. *Am. J. Clin. Pathol.*, **78**, 806.

BREMMER, R.D., NISBET, A.D., HERRIOT, R. & 4 others. (1981). Detection of placental protein five (PP5) and pregnancy-specific glycoprotein ($SP_1$) in benign and malignant breast disease. *Oncodev. Biol. Med.*, **2**, 55.

GRUDZINSKAS, J.G., COOMBES, R.C., RATCLIFFE, J.G. & 4 others. (1980). Circulating levels of pregnancy specific $\beta_1$ glycoprotein in patients with testicular, bronchogenic and breast carcinomas. *Cancer*, **45**, 102.

HEYDERMAN, E. (1979). Immunoperoxidase technique in histopathology: applications, methods and controls. *J. Clin. Pathol.*, **32**, 971.

HORNE, C.H.W., REID, I.N. & MILNE, G.D. (1976). Prognostic significance of inappropriate production of pregnancy proteins by breast cancers. *Lancet*, **ii**, 279.

HUNTER, W.M. & BENNIE, J.G. (1979). Reduction of non-specific serum responses in human pituitary gonadotrophin radioimmunoassays. *J. Endocrinol.*, **80**, 59.

KAMINSKA, J., CALVERT, I. & ROSEN, S.W. (1979). Radioimmunoassay of "pregnancy-specific" $\beta_1$-glycoprotein ($SP_1$). *Clin. Chem.*, **25**, 577.

INABA, N., RENK, T., WURSTER, K., RAPP, W. & BOHN, H. (1980). Ectopic synthesis of pregnancy specific $\beta_1$-glycoprotein ($SP_1$) and placental specific tissue proteins ($PP_5$, $PP_{10}$, $PP_{11}$, $PP_{12}$) in nontrophoblastic malignant tumours. Possible markers in oncology. *Klin Wochenschr.*, **58**, 789.

ROSEN, S.W., GAIL, M.H. & TORMEY, D.C. (1982). Use of circulating pregnancy specific $\beta_1$-glycoprotein as a marker in carcinoma of the breast in women. *J. Natl Cancer Inst.*, **69,** 1067.

REINSCH, C.H. (1967). Smoothing by spline functions. *Num. Math.*, **10,** 177.

SEARLE, F., LEAKE, B.A., BAGSHAWE, K.D. & DENT, J. (1978). Serum $SP_1$-pregnancy-specific-$\beta$-glycoprotein in choriocarcinoma and other neoplastic disease. *Lancet,* **i,** 579.

SØRENSEN, S. & TRENTEMØLLER, S. (1983). Purification of the major component of the pregnancy specific $\beta_1$-glycoprotein ($SP_1$) by affinity chromatography, and application to a highly sensitive radioimmunoassay. *Oncodev. Biol. Med.*, **4,** 351.

TATARINOV, Y.S. (1980). The diagnostic value of circulating trophoblast-specific $\beta_1$-glycoprotein (TSG) in cancer patients. *Br. J. Cancer,* **41,** 821.

WALKER, R.A. (1981). Differentiation of human breast carcinomas: An immunohistological study of appropriate and inappropriate protein production. *J. Pathol.*, **135,** 87.

WÜRZ, H. (1979). Serum concentrations of $SP_1$ (pregnancy-specific-$\beta_1$-glycoprotein) in healthy, nonpregnant individuals, and in patients with nontrophoblastic malignant neoplasms. *Arch. Gynecol.*, **227,** 1.